# Genome-Wide Identification of Sigma Factors in *Brassica napus* and Role of *BnSIG5A* in Response to Cold Stress

**DOI:** 10.3390/ijms27073010

**Published:** 2026-03-26

**Authors:** Yiwa Hu, Yingying Zhou, Iram Batool, Wenqiang Lan, Qian Huang, Basharat Ali, Muhammad Arslan Yousaf, Kangni Zhang, Jiali Ma, Ahsan Ayyaz, Weijun Zhou

**Affiliations:** 1 Ministry of Agriculture and Rural Affairs Key Laboratory of Spectroscopy Sensing, Institute of Crop Science, Zhejiang University, Hangzhou 310058, China; 2 Department of Agricultural Engineering, Khwaja Fareed University of Engineering and Information Technology, Rahim Yar Khan 64200, Pakistan; 3 Key Laboratory of Plant Secondary Metabolism and Regulation of Zhejiang Province, College of Life Sciences and Medicine, Zhejiang Sci-Tech University, Hangzhou 310018, China

**Keywords:** gene family, identification, sigma factor, cold stress, functional characterization, photosynthesis

## Abstract

Sigma factors (SIGs) are nuclear-encoded regulators of chloroplast gene transcription. We conducted a genome-wide analysis in *Brassica napus*, identifying 23 SIG genes that were phylogenetically classified into six distinct subfamilies. Characterization of gene structure, conserved motifs, and chromosomal locations indicated family expansion primarily through segmental duplication under purifying selection. Promoter analysis identified cold-responsive elements enriched in *BnSIG5A*. Expression profiling showed that *BnSIG5* subfamily members, particularly *BnSIG5A*, are strongly induced by cold stress. Analysis of *Arabidopsis* SIG5 mutants confirmed previously reported roles of AtSIG5 in cold tolerance. Heterologous expression in yeast, and the strong cold induction of *BnSIG5A* together with its chloroplast localization, suggest that *BnSIG5A* may play a conserved role, providing a foundation for future functional studies in *B. napus*. This work establishes a genomic framework for the SIG family in rapeseed and identifies *BnSIG5A* as a high-priority candidate for further investigation. Subcellular localization confirmed chloroplast targeting of BnSIG5A. Heterologous expression in yeast and analysis of *Arabidopsis* SIG5 mutants suggest conserved functions in cold tolerance, providing a foundation for future functional studies in *B. napus*. This work establishes a genomic framework for understanding SIG-mediated stress responses in rapeseed and identifies *BnSIG5A* as a promising candidate for further investigation.

## 1. Introduction

*Brassica napus* (oilseed rape) is a fundamental crop in global agriculture, serving as a primary source of vegetable oil and biofuel. As the leading oilseed crop in China, it accounts for roughly 20% of worldwide production, cultivated on over 7.5 million hectares [1]. The productivity of this vital crop, however, is consistently threatened by abiotic stresses [2]. Among these stressors, low temperature is a major environmental constraint that severely impairs growth, development, and yield, ultimately shaping its geographic distribution. Understanding the molecular mechanisms underlying cold adaptation in *B. napus* is crucial for ensuring agricultural stability.

Chloroplasts are central to a plant’s ability to perceive and respond to environmental challenges like cold stress. These organelles possess their own genome, transcribed by a plastid-encoded RNA polymerase (PEP). The PEP complex, a bacteria-like multi-subunit RNA polymerase, requires nuclear-encoded sigma factors (SIGs) for promoter recognition. *Sigma factor* genes, which were evolutionarily transferred to the nucleus, are essential components of retrograde signaling pathways that modulate nuclear gene expression in response to plastid status [3,4,5,6]. Structurally, plant *SIGs* belong to the sigma-70 family and are predicted to localize to chloroplasts [7,8,9]. They share conserved C-terminal domains (1.2, 2, 3, and 4) with their bacterial homologs, which are responsible for core promoter recognition and transcription initiation [10,11]. In contrast, their N-terminal regions are more variable, suggesting functional diversification in plants [11,12,13]. Through this mechanism, *SIGs* are indispensable for chloroplast development, chlorophyll biosynthesis, and the regulation of photosynthetic genes, thereby directly influencing plant productivity and stress resilience [14,15,16].

The functionality of chloroplasts is intimately linked to stress tolerance. Cold stress disrupts critical chloroplast processes, including photoprotection, thylakoid membrane integrity, and reactive oxygen species (ROS) homeostasis, ultimately increasing excitation pressure on photosystem II (PSII) [4,13,17,18]. This positions SIGs as critical regulators of the chloroplast’s response to environmental adversity. In angiosperms, six phylogenetically distinct *SIG* groups (*SIG1*–*SIG6*) have been identified [19]. Evolutionary analyses, based on conserved intron positions, suggest that *SIG2*, *SIG3*, *SIG4*, and *SIG6* originated from a common ancestor, while *SIG1* and *SIG5* have followed a more divergent evolutionary path [4,12].

In the model plant *Arabidopsis thaliana*, the six sigma factors (*SIG1*–*SIG6*) regulate chloroplast transcription during both biogenesis and steady-state photosynthesis [17,20,21,22]. Notably, *SIG5* has emerged as a key player in mediating chloroplast responses to diverse stimuli, including fluctuating light conditions, abiotic stresses, and circadian signals [23,24,25]. This makes *SIG5* a particularly compelling candidate for investigating stress response pathways, especially cold stress [2]. Despite this knowledge, a comprehensive analysis of the SIG gene family in the complex allotetraploid genome of *B. napus* and its specific role in cold tolerance remains largely unexplored.

While the function of the SIG gene family has been well-characterized in *Arabidopsis thaliana* [2,20,23,24], including its role in integrating cold and circadian signals [2], the SIG genes in the economically vital crop *B. napus* remains unexplored. *B. napus* is an allotetraploid species (AACC genome) resulting from hybridization between *B. rapa* (AA) and *B. oleracea* (CC), with a genome approximately four times larger than *Arabidopsis*. This polyploidization event has led to gene duplication and potential functional diversification, making it impossible to simply extrapolate findings from diploid model species. Understanding how SIG genes, particularly *SIG5* paralogs, function in this complex genomic context is essential for two reasons: (1) It reveals how essential regulatory networks evolve and partition functions following whole-genome duplication; and (2) it provides crop-specific knowledge required for genetic improvement of cold tolerance in *B. napus*, which is cultivated across diverse climatic zones.

This study aimed to fill this knowledge gap by conducting a genome-wide identification and characterization of the SIG gene family in *B. napus*. We analyzed the sequence features, phylogenetic relationships, and chromosomal distribution of *BnSIG* genes. By integrating transcriptome data, we systematically profiled their expression patterns under cold stress, which highlighted *BnSIG5A* as a strong cold-inducible candidate. To gain insight into potential SIG5 function, we examined the response of *Arabidopsis* T-DNA insertion mutants (*sig5-3* and *sig5-7*) to cold stress, alongside heterologous expression of *BnSIG5A* in yeast. While these approaches cannot substitute for direct functional validation in *B. napus*, they provide suggestive evidence for conserved *SIG5* function across species. Our findings provide a comprehensive genomic framework for the *BnSIG* family and identify *BnSIG5A* as a strong candidate for future functional studies aimed at the genetic improvement of cold tolerance in *B. napus*.

## 2. Results

### 2.1. Genome-Wide Identification and Evolutionary Characterization of the BnSIG Gene Family

A comprehensive genome-wide analysis identified 23 sigma factor (*SIG*) genes in *B. napus*. These genes were systematically characterized and named *BnSIG1A* to *BnSIG6D* based on their phylogenetic relationships. The analysis encompassed their physio-chemical properties, evolutionary history, and structural features, revealing insights into the family expansion and functional conservation.

#### 2.1.1. Identification and Physio-Chemical Properties of BnSIG Proteins

The identified 23 *SIG* genes in the *B. napus* genome encoded proteins exhibiting considerable diversity in length (88–684 amino acids) and molecular weight (10.33–77.54 kDa). This substantial variation reflects evolutionary processes including domain gain/loss, insertion/deletion events, and N-/C-terminal variation [26]. Notably, the shorter variants (e.g., BnSIG1A at 88 aa) lack some conserved domains present in full-length family members (Supplementary Table S1), suggesting possible neo-functionalization or alternative regulatory roles. In contrast, full-length variants (e.g., BnSIG3A at 684 aa) retain all typical sigma factor domains required for core RNA polymerase binding and promoter recognition. This structural diversity within a single gene family enables functional specialization while maintaining core conserved functions under purifying selection [27,28]. Most BnSIG proteins (22 out of 23) were predicted to be basic (pI > 8), with only BnSIG2B being acidic (pI 6.47) (Supplementary Table S1). Hydropathicity analysis indicated that all BnSIGs are hydrophilic, with instability indices exceeding 40, classifying them as unstable proteins; BnSIG4D was the most unstable (index = 78.89) and BnSIG6A the least (index = 41.60) (Supplementary Table S1).

Subcellular localization predictions placed BnSIG proteins in multiple compartments, including the chloroplast, cytoplasm, and nucleus, though the chloroplast was the predominant predicted location, consistent with their functional role (Supplementary Table S2). Secondary structure analysis indicated that α-helices and random coils are the primary structural components of all BnSIG proteins, with no significant β-sheet or β-turn structures predicted (Supplementary Table S2).

#### 2.1.2. Phylogenetic Relationships, Chromosomal Distribution, and Collinearity Analysis

**Figure 1:**
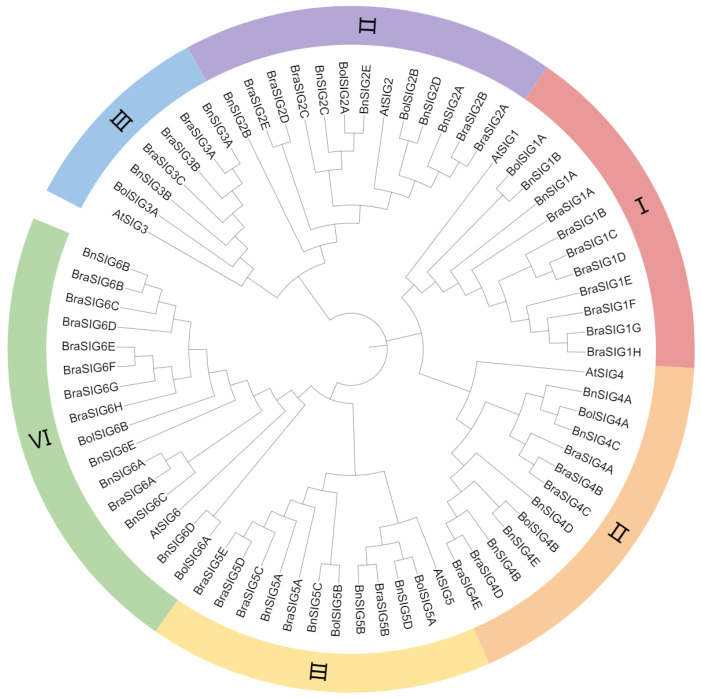
Phylogenetic tree of *SIG* gene family members among *B. rapa*, *B. napus*, *B. oleracea* and *A. thaliana*.

A maximum-likelihood phylogenetic tree was constructed using 73 SIG protein sequences from *A. thaliana* (6), *B. rapa* (34), *B. oleracea* (10), and *B. napus* (23). The analysis classified the SIG family into six well-supported clades (Groups I-VI) (Figure 1). Group VI was the largest, while Group III was the smallest, suggesting differential expansion and diversification of the family across these species.

**Figure 2:**
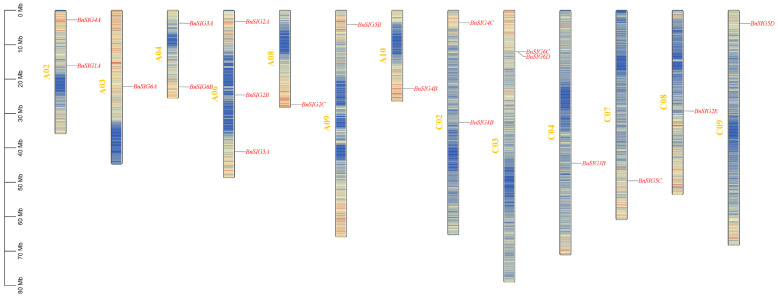
Chromosomal location of *BnSIG* gene family members in *B. napus*.

The 23 *BnSIG* genes were unevenly distributed across 13 of the 19 *B. napus* chromosomes (A02, A03, A04, A06, A08, A09, A10, C02, C03, C04, C07, C08, and C09) (Figure 2). Chromosome A06 harbored the most genes (three), while eight chromosomes contained only a single *BnSIG* gene. This irregular distribution implies that segmental duplication events contributed significantly to the family expansion.

**Figure 3:**
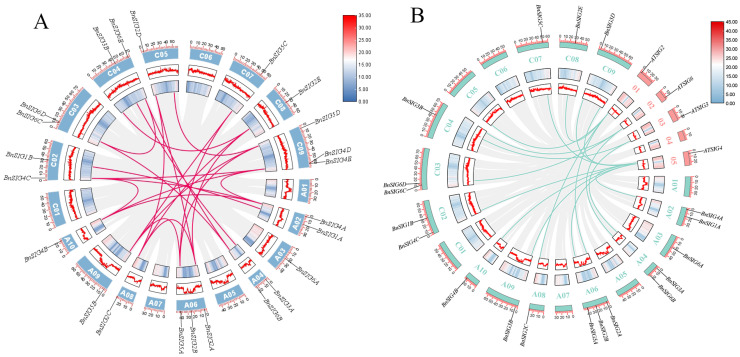
(**A**) Collinearity relationship of *SIG* gene family members in *B. napus*; (**B**) homology analysis of *SIG* gene between *B. napus* and *A. thaliana*. The red rectangles indicate *A. thaliana* chromosomes, and the green rectangles signify *B. napus* chromosomes.

Collinearity analysis within the *B. napus* genome identified 17 duplicated gene pairs, underscoring the role of segmental duplication in the proliferation of the *SIG* family (Figure 3A). Key genes like *BnSIG1A*, *BnSIG4A*, *BnSIG4B*, and *BnSIG6A* were involved in multiple duplication events. Notably, *BnSIG5C* showed collinearity with both *BnSIG5A* and *BnSIG5B*, suggesting a potential triplication event. Analysis of non-synonymous (Ka) and synonymous (Ks) substitution rates for these pairs revealed Ka/Ks ratios all less than one, indicating the action of strong purifying selection (Supplementary Table S3). Further collinearity analysis between *B. napus* and *A. thaliana* identified 17 orthologous relationships involving 14 *BnSIG* and four *AtSIG* genes (e.g., *AtSIG2* with *BnSIG2A*, *BnSIG2C*, and *BnSIG2E*), all of which belonged to the same phylogenetic subfamilies (Figure 3B). This highlights a high degree of evolutionary conservation following polyploidization.

#### 2.1.3. Gene Structure and Conserved Motif Analysis of BnSIG Proteins

**Figure 4:**
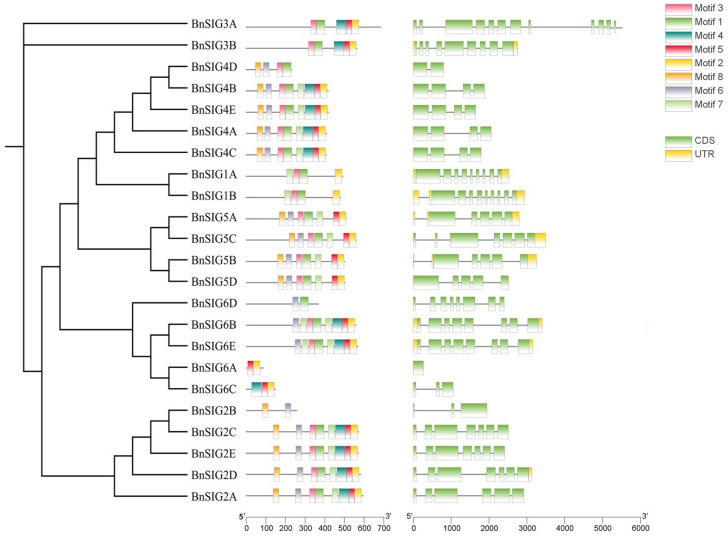
Evolutionary relationship, gene structure and distribution of conserved motifs of *SIG* gene family members in *B. napus*.

Analysis of gene structure revealed that most *BnSIG* genes contain a simple structure of one to three exons, though *BnSIG3A* was more complex with twelve exons (Figure 4). Most genes (14 out of 23) lacked predicted 5′ and 3′ UTRs in the current genome annotation, which likely reflects incomplete annotation of untranslated regions rather than true biological absence. Genes within the same phylogenetic clade generally shared similar exon-intron structures, supporting their evolutionary relatedness.

Conserved motif analysis identified ten motifs, with most BnSIG proteins sharing a common set, indicating structural conservation within the family (Figure 4). However, notable variation in motif composition was observed among specific members. For instance, BnSIG1A, BnSIG4C, and BnSIG5C retained only two of the ten motifs, while other family members retained most or all motifs. This variation in conserved motif content may reflect genuine structural differences, though it could also result from incomplete genome annotation or gene model prediction errors—a common limitation in analyses of recently sequenced polyploid genomes [28]. Determining whether such structural variation translates to functional differences will require experimental investigation.

### 2.2. Regulatory and Expression Characteristics of BnSIG Genes

To understand the regulatory potential and biological roles of the *BnSIG* family, we analyzed promoter *cis*-elements, expression patterns, and subcellular localization. These analyses highlighted the importance of specific members, particularly *BnSIG5A*, in stress and light responses.

#### 2.2.1. Promoter Cis-Element Analysis

**Figure 5:**
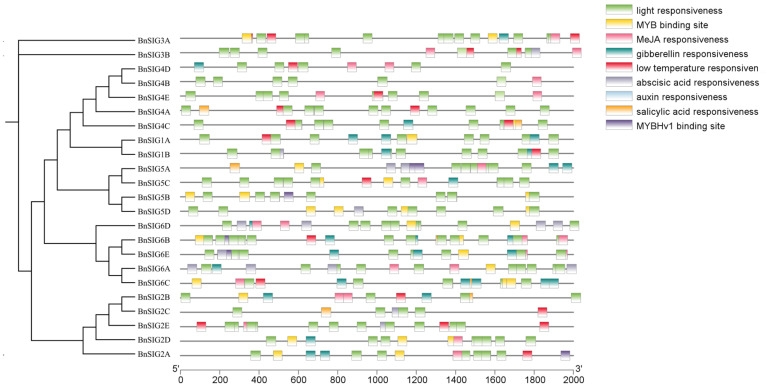
Cis-acting elements of *B. napus SIG* gene family. *In silico* prediction of cis-acting regulatory elements in the 2000 bp upstream promoter regions of *BnSIG* genes using the PlantCARE database. Elements were identified based on sequence homology to known motif patterns and categorized by predicted function. These predictions are computational and require experimental validation to confirm actual regulatory activity.

Analysis of the 2.0 kb promoter regions upstream of all *BnSIG* genes revealed the presence of multiple predicted cis-acting regulatory elements associated with stress and hormone responses (Figure 5). These predictions are based on sequence homology to known element motifs in the PlantCARE database and should be interpreted as suggestive rather than demonstrative of actual regulatory function. Notably, putative low-temperature-responsive elements (LTR) were identified in 16 promoters, and MYB binding site motifs—associated with drought and cold stress responses in other species—were also detected, with *BnSIG5A* containing four such motifs. Additionally, all *BnSIG* promoters contained predicted light-responsive elements, consistent with the established connection between sigma factors and photosynthetic gene regulation. These in silico predictions generate testable hypotheses regarding the regulation of *BnSIG* genes by light and cold stress, which require experimental validation through techniques such as promoter-reporter assays, electrophoretic mobility shift assays (EMSAs), or chromatin immunoprecipitation (ChIP).

#### 2.2.2. Expression Profiling Across Tissues and Under Cold Stress

**Figure 6:**
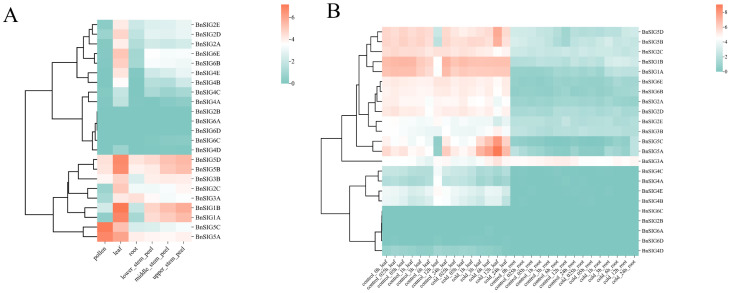
(**A**) Tissue-specific expression pattern analysis of *BnSIGs*. Different color changes illustrate the log2-transformed FPKM + 1, where orange, white, and green colors present high to low levels of expression. (**B**) Analysis of the *BnSIG* genes’ expression under cold stress (4 °C). Different colors illustrate the log2-transformed FPKM + 1, where orange signifies greater levels of expression and green reflects low expression levels.

Transcriptomic data revealed that *BnSIG* genes exhibit distinct tissue-specific expression patterns (Figure 6A). Members of the *BnSIG5* subfamily (*BnSIG5A*, *BnSIG5B*, *BnSIG5D*) were highly expressed across most tissues, including pollen and leaves. In contrast, genes like *BnSIG2B* and *BnSIG6A* showed low or undetectable expression. Under cold stress, a dynamic transcriptional response was observed in leaves (Figure 6B). *BnSIG5A*, *BnSIG5B*, and *BnSIG5C* were strongly induced. *BnSIG5A* exhibited a rapid response, with significant upregulation beginning at 1 h, peaking at 2 h, and remaining elevated until 12 h post-treatment, marking it as a key early responder to cold stress.

#### 2.2.3. Subcellular Localization of BnSIG5A

**Figure 7:**
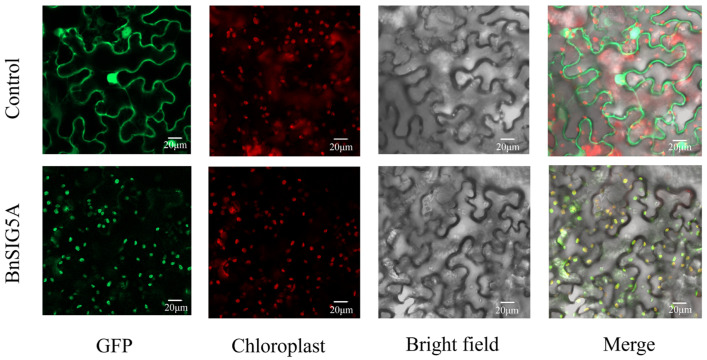
The subcellular locations of BnSIG5A protein in *Nicotiana benthamiana* leaves.

The subcellular localization of BnSIG5A was confirmed experimentally. Transient expression of a BnSIG5A-GFP fusion protein in *Nicotiana benthamiana* leaves resulted in a GFP signal that co-localized with chloroplasts, validating its plastid targeting and functional role in chloroplast transcription (Figure 7).

### 2.3. Expression Analysis of BnSIG Genes and Functional Insights from Arabidopsis SIG5 Mutants

To investigate the cold-responsive expression of *BnSIG* genes in *B. napus*, we performed qRT-PCR analysis on selected family members. To examine whether the *Arabidopsis* ortholog of *BnSIG5A* (*AtSIG5*) functions in cold tolerance, we analyzed *A. thaliana SIG5* T-DNA insertion mutants. These experiments test the function of *AtSIG5* and provide a reference point for hypotheses regarding potential conserved roles of *BnSIG5A*.

#### 2.3.1. qRT-PCR Validation of Cold-Responsive *BnSIG* Genes

**Figure 8:**
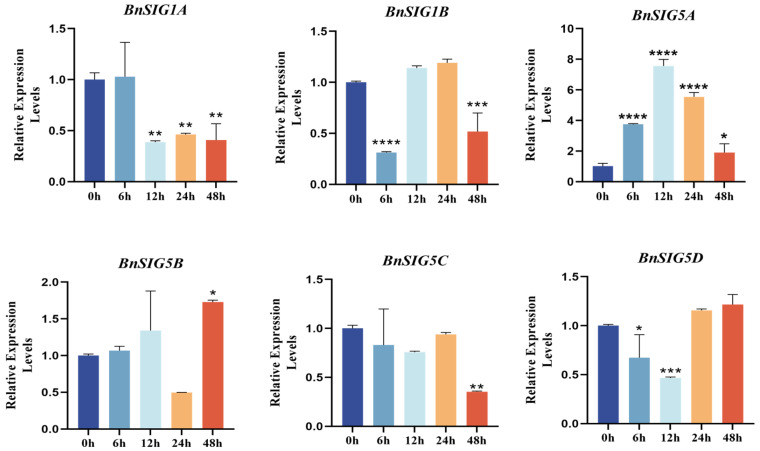
Expression analysis of *BnSIGs* under cold treatments by RT-qPCR. The 2^−ΔΔCt^ method was employed to assess gene expression patterns relative to actin, based on three replicates. The results are presented as the mean ± standard deviation. Statistical significance was evaluated using Student’s t-test; 0 h serves as control, with significance levels indicated as follows: * = 0.05, ** = 0.01, *** = 0.001, **** = 0.0001.

The qRT-PCR analysis of six selected *BnSIG* genes confirmed the RNA-seq expression trends (Figure 8). *BnSIG5A* expression peaked sharply at 12 h of cold treatment, while *BnSIG5B*, *BnSIG5C*, and *BnSIG5D* showed sustained induction up to 24 h. In contrast, *BnSIG1A* expression declined steadily, suggesting it is not a primary cold-responsive gene. These results identify the *BnSIG5* subfamily, particularly *BnSIG5A*, as strong cold-inducible candidates in *B. napus*.

#### 2.3.2. Low-Temperature-Tolerance Assays of Yeast Transformants

**Figure 9:**
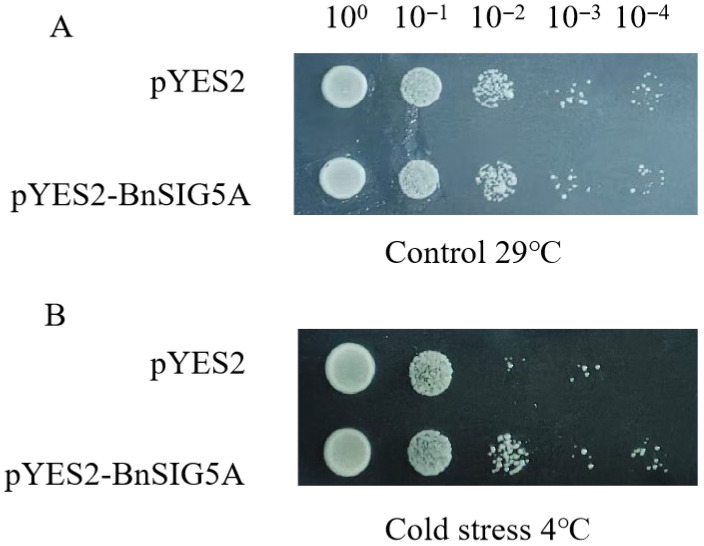
Overexpression of *BnSIG5A* enhances tolerance to cold stress in transformant yeast (*BY4741*). (**A**) Transformant yeast was grown in 29 °C, 48 h. (**B**) Transformant yeast was grown in 4 °C, 48 h.

As a heterologous system to assess whether BnSIG5A protein expression could confer stress tolerance in a simple eukaryotic context, we transformed yeast (*Saccharomyces cerevisiae* strain BY4741) with either a pYES2-*BnSIG5A* vector or an empty vector control. Under optimal growth conditions (29 °C), both transformed strains showed comparable colony growth. However, when subjected to cold stress (4 °C for 48 h), the *BnSIG5A*-expressing yeast exhibited enhanced growth compared to the empty vector control (Figure 9). While yeast lacks plastids and cannot support sigma factor transcriptional function, this result suggests that *BnSIG5A* expression may confer cold tolerance through mechanisms independent of its native transcriptional role, such as enhanced protein stability or general stress protection, or may simply reflect successful heterologous protein production. This experiment provides complementary, albeit indirect, evidence that *BnSIG5A* can be functionally expressed in a eukaryotic system but does not demonstrate sigma factor-specific activity.

#### 2.3.3. Phenotypic and Physiological Analysis of *A. thaliana sig5* Mutants

**Figure 10:**
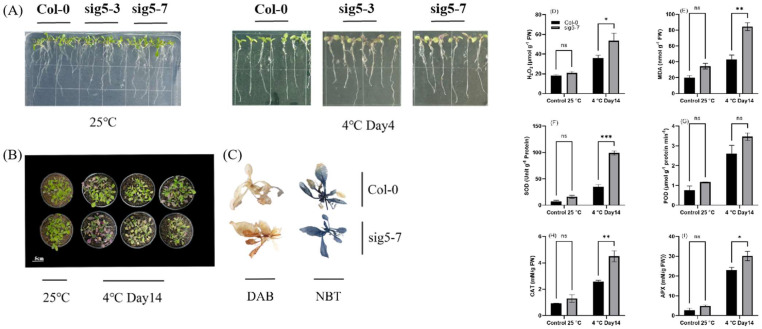
Comparative phenotype of *A. thaliana* T-DNA insertion mutant lines in leaves and roots under cold stress. (**A**) The left indicates one-week seedlings of Col-0, *sig5-3*, and *sig5-7* under normal conditions; the right were subjected to cold treatment of 4 °C for 4 days. (**B**) The left indicates 20-day-old seedlings of Col-0 and *sig5-7* under normal conditions; the right were subjected to cold treatment of 4 °C for 14 days. (**C**) Histochemical staining images show the ROS production (H_2_O_2_ and O_2_^−^) in the leaves of both genotypes under cold treatment. DAB staining indicates the accumulation of H_2_O_2_, while NBT staining indicates the accumulation of O_2_^−^. (**D**) hydrogen peroxide (H_2_O_2_), (**E**) malondialdehyde (MDA), antioxidant enzyme activities, (**F**) superoxide dismutase (SOD), (**G**) peroxidase (POD), (**H**) catalase (CAT), and (**I**) ascorbate peroxidase (APX), respectively. Different letters represent significance level as * = 0.05, ** = 0.01, *** = 0.001, and ns = non-significance.

To further functionally characterize *SIG5,* we analyzed two *A. thaliana* T-DNA insertion mutants, *sig5-3* and *sig5-7*. Under cold stress, both mutants exhibited a clear susceptible phenotype, including leaf browning, which was absent in the wild type (Col-0) (Figure 10A,B).

Physiological assessments revealed that the *sig5-7* mutant accumulated significantly higher levels of hydrogen peroxide (H_2_O_2_) and malondialdehyde (MDA) under cold stress, indicating severe oxidative damage (Figure 10 D,E). This was visually confirmed by intense DAB and NBT staining (Figure 10C). Although the activities of antioxidant enzymes (SOD, POD, CAT and APX) were elevated in the mutant, this response was insufficient to prevent oxidative damage.

**Figure 11:**
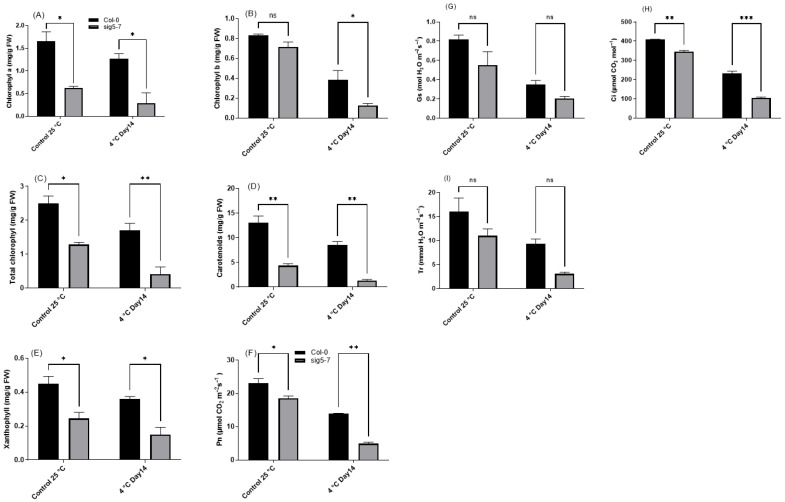
Photosynthetic pigments in *sig5-7* mutant under cold stress as compared to Col-0. (**A**) Chlorophyll a, (**B**) chlorophyll b, (**C**) total chlorophyll, (**D**) carotenoids, and (**E**) xanthophyll, respectively. Gas exchange parameters in *sig5-7* mutant under cold stress as compared to Col-0. (**F**) Net photosynthesis (Pn), (**G**) stomatal conductance (Gs), (**H**) carbon dioxide intake (Ci), and (**I**) transpiration rate (Tr), respectively. Different letters represent significance level as * = 0.05, ** = 0.01, *** = 0.001, and ns = non-significance.

**Figure 12:**
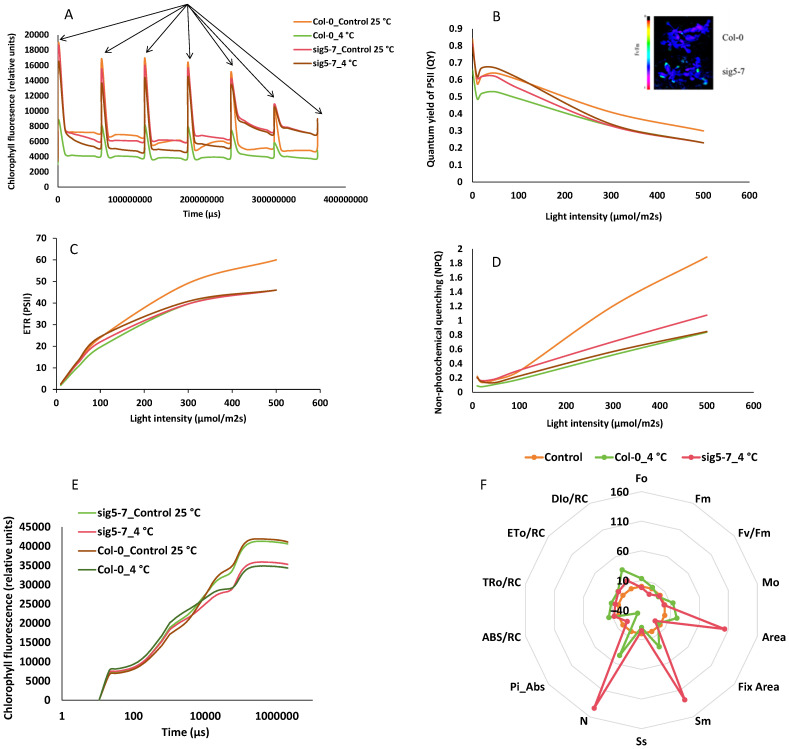
Relative values of chlorophyll fluorescence in *sig5-7* mutant under cold stress as compared to Col-0 (**A**). (**B**) Quantum yield of PSII (QY), (**C**) electron transport rate (ETR), (**D**) non-photochemical quenching (NPQ), (**E**) OJIP test relative values, and (**F**) radar plot of computed values for OJIP test of PSI, respectively.

The mutants also displayed significantly reduced chlorophyll a, b, and total chlorophyll content, as well as impaired gas exchange parameters (Pn, Gs, Ci, Tr) compared to Col-0, both before and after cold stress (Figure 11). Chlorophyll fluorescence analysis showed that the maximum quantum efficiency of PSII (Fv/Fm) was lower in the mutant after prolonged cold treatment (Figure 12). However, the mutant showed an altered OJIP (the analysis of the fast chlorophyll fluorescence rise curve through its O, J, I, and P steps) curve and higher non-photochemical quenching (NPQ), suggesting a compensatory mechanism to manage excess excitation energy. Despite this, the overall data demonstrate that the loss of *SIG5* function compromises PSII integrity and photosynthetic performance under cold stress. To assess the impact of the mutation on gene expression related to RuBP hydroxylase large subunit synthesis [29] and the psbI-psbK-psbD-psbC [17,30,31], we performed quantitative reverse transcription PCR (qRT-PCR) analysis. Compared with the wild type, the expression levels of these genes were consistently downregulated in the mutant, with the most pronounced reduction observed for psbD (Supplementary File S1). Collectively, these results confirm that *SIG5* is essential for cold tolerance, playing a critical role in mitigating oxidative stress, preserving chlorophyll content, and maintaining photosynthetic efficiency.

## 3. Discussion

Plastid sigma factors (*SIGs*) serve as master regulators of chloroplast transcription, enabling the nucleus to dynamically control plastid function in response to developmental and environmental signals. Our study provides the first comprehensive genomic and functional characterization of the *SIG* gene family in the allotetraploid crop *B. napus*, with a specific focus on its role in cold stress adaptation. We demonstrate that the *BnSIG* family has expanded through polyploidization, that specific members are potent cold-stress responders, and that *BnSIG5A* is essential for maintaining photosynthetic integrity and oxidative homeostasis under low-temperature stress.

### 3.1. Genomic Expansion and Functional Diversification of the BnSIG Family

The identification of 23 *SIG* genes in *B. napus* reflects the genomic complexity of this allotetraploid species. Notably, while *Arabidopsis* contains a single *SIG5* gene, *B. napus* possesses four *SIG5* homologs (*BnSIG5A-D*). This expansion raises important questions about functional partitioning: do all four paralogs retain the same function, or have they undergone sub-functionalization or neo-functionalization following polyploidization? Our expression data show that all four *BnSIG5* paralogs are cold-inducible, but with distinct temporal dynamics (Figure 8), suggesting possible divergence in regulatory control. This represents a key biological difference between the model system and the crop species, highlighting the necessity of species-specific studies.

The present work provides the foundational genomic resources needed to address such questions and ultimately enables targeted manipulation of individual paralogs for crop improvement. The observed protein length variation (88–684 aa) within the BnSIG family warrants discussion about its biological significance. This diversity is not random but reflects evolutionary processes shaping functional diversification following whole-genome duplication in *B. napus* [26]. Similar length variation has been documented in other plant gene families and is associated with sub-functionalization and neo-functionalization [27].

The shorter variants, such as BnSIG1A (88 aa) and BnSIG4C (147 aa), lack several conserved C-terminal domains (regions 2–4) typically required for core RNA polymerase binding and promoter recognition (Figure 4). These truncated forms may represent: (1) Pseudogenes undergoing decay, (2) alternative splice variants with regulatory functions, or (3) proteins that have acquired novel functions independent of canonical sigma factor activity. In contrast, full-length variants like BnSIG3A (684 aa) retain all conserved domains and likely function as canonical sigma factors in chloroplast transcription.

Notably, all four BnSIG5 paralogs are full-length proteins (541–568 aa), suggesting they retain canonical sigma factor function. This is consistent with their strong cold-inducible expression and predicted role in chloroplast gene regulation. The presence of multiple paralogs with full-length sequences in the SIG5 clade, compared to truncated forms in other clades, suggests that SIG5 function is under stronger purifying selection and that all four copies remain functionally important in *B. napus*.

This structural diversity within a single gene family exemplifies how polyploid genomes generate functional novelty through gene duplication and divergence, providing raw material for environmental adaptation. The length variation we observe likely contributes to the regulatory flexibility required for *B. napus* to cope with diverse environmental stresses, including cold.

The phylogenetic classification of BnSIG proteins into six conserved clades aligns with established groupings in *A. thaliana* and rice, indicating deep evolutionary conservation of the SIG family’s core structure in angiosperms [32,33,34,35]. Of the six *Arabidopsis* sigma subunits, each plays a distinct role in regulating chloroplast gene expression. *SIG1* regulates the transcription of the photosystem reaction center genes psaA/B and psbA, with its phosphorylation being influenced by the redox status of the plastoquinone pool [36]. *SIG2*, on the other hand, is involved in the transcription of several chloroplast tRNA genes, including trnE, which encodes tRNA-Glu, and may link translation with pigment synthesis in chloroplasts [17]. *SIG3*, a nucleus-encoded plastid sigma factor, specifically transcribes the psbN gene in plastids, playing a crucial role in chloroplast gene expression [37]. In the unicellular red alga *Cyanidioschyzon merolae*, the nuclear-encoded sigma factor *SIG4* directly activates the transcription of chloroplast genes psbA and ycf17, both of which are important for photosynthesis and plastid function. Research on *AtSIG6*, another plastid sigma factor from Arabidopsis, highlights how cpCK2 phosphorylation modulates its function in regulating chloroplast gene expression [11]. Finally, *SIG5* is stress-induced and contributes to the repair of damaged photosystem II (PSII) by transcribing the psbD and psbC genes [17]. Together, these findings help to clarify the target genes and physiological roles of each sigma subunit in plant cells.

The uneven distribution of *BnSIG* genes across chromosomes and the prevalence of segmental duplication events strongly suggest that genome duplication has been a primary driver for the expansion of this gene family in *B. napus*. The finding that all duplicated *BnSIG* pairs have undergone purifying selection (Ka/Ks < 1) indicates strong functional constraints, preserving their essential roles in chloroplast biology despite genomic rearrangement [32]. Further evidence of this balance between conservation and diversification is found in the gene structure and motif composition. Members within the same phylogenetic clade share highly similar exon-intron structures and conserved motifs, suggesting functional redundancy. However, the selective loss of motifs in specific members, such as *BnSIG2B*, *BnSIG6A*, *BnSIG6D*, and *BnSIG6C*, points to potential sub- or neo-functionalization. This structural divergence may explain the specialized roles that different *SIG* paralogs have acquired in the complex regulatory networks of *B. napus*.

### 3.2. Expression Patterns and Potential Regulatory Roles Under Cold Stress

The expression of *SIG* genes is potentially influenced by cis-acting elements in their promoters. Our *in silico* promoter analysis identified abundant light-responsive element motifs in all *BnSIG* promoters (Figure 3A), which correlates with previous findings in maize and *A. thaliana* where *SIG* genes such as *ZmSIG1* and *AtSIG5* are light-inducible and involved in photomorphogenesis and high-light responses [38,39,40]. This correlation suggests a possible conserved relationship between sigma factors and light-regulated photosynthetic gene expression, though direct experimental validation is required to confirm that these predicted motifs are functionally active.

Importantly, our analysis also identified putative low-temperature responsive (LTR) and MYB cis-element motifs in multiple *BnSIG* promoters, with *BnSIG5A* showing a particularly high density of such elements (Figure 3A). These predictions align with the observed rapid and strong transcriptional upregulation of *BnSIG5A* under cold stress (Figure 6 and Figure 8), and are consistent with the cold-induction of *AtSIG5* reported in *Arabidopsis* [2,20]. The convergence of light and cold stress-related element predictions on the *SIG5* promoter across species raises the hypothesis that a conserved regulatory mechanism coordinates photosynthetic capacity with abiotic stress responses. However, definitive evidence for such regulation, such as demonstration of transcription factors binding to these elements, or functional validation through promoter-reporter assays remains to be established.

The subsequent confirmation of BnSIG5A chloroplast localization (Figure 7) supports its potential role in mediating nuclear-plastid communication under environmental stress, but does not itself demonstrate regulatory function. Together, these predictive and correlative findings generate specific, testable hypotheses regarding the regulation of *BnSIG* genes by light and cold stress, providing a foundation for future experimental validation studies.

### 3.3. Insights into BnSIG5A Function from Arabidopsis thaliana T-DNA Insertion Mutants

To explore the potential function of *SIG5* in chloroplast resilience, we examined the cold stress response of *Arabidopsis thaliana* T-DNA insertion mutants (*sig5-3* and *sig5-7*). *Arabidopsis* is a well-established model plant with mature genetic tools, and its *SIG5* gene shares high homology with *BnSIG5A*, providing a reliable reference for investigating *SIG5* function, following previously established study [2]. Consistent with recent reports documenting *SIG5*’s role in integrating cold and circadian signals [2], both mutant lines exhibited cold-sensitive phenotypes, including leaf browning, elevated ROS accumulation, and significant chlorophyll loss under low-temperature stress (Figure 5 and Figure 6). These observations align with the photoprotective role of *SIG5* described in earlier studies [20] and confirm that *SIG5* function in promoting chloroplast resilience is conserved in *Arabidopsis*. Based on the experimental results in *Arabidopsis thaliana*, we hypothesize that *BnSIG5A* may play a similar role in chloroplast resilience and cold tolerance. However, direct physiological evidence for *BnSIG5A* in *B. napus* was not obtained in this study, and these conclusions are derived from extrapolation of the *Arabidopsis* mutant data. The impaired PSII efficiency (reduced Fv/Fm) and altered chlorophyll fluorescence kinetics (OJIP parameters) in the *sig5* mutant directly link the loss of *SIG5* function to a failure in photoprotection. These findings are consistent with studies showing that proper *SIG* function is crucial for maintaining electron transport rates and preventing over-reduction in the photosynthetic electron transport chain under stress conditions [21]. In *A. thaliana*, *AtSIG5* is known to directly activate the transcription of the *psbD* gene, which encodes the D2 protein of PSII, and this regulation is enhanced by cold stress via the HY5/HYH pathway [2,20]. The photosynthetic defects observed in *sig5* mutants under cold stress are therefore consistent with disrupted *psbD* expression and impaired PSII repair.

The disruption in photosynthetic electron transport observed in *sig5* mutants likely contributes to the ROS imbalance (Figure 5). Under cold stress, the impaired linear electron flow leads to enhanced leakage of electrons to O_2_, generating superoxide radicals primarily at PSI [35]. Our observation of increased superoxide accumulation in *sig5* mutants (NBT staining, Figure 10C) aligns with this mechanism, and the subsequent conversion to H_2_O_2_ (DAB staining, Figure 10C) is consistent with cold-induced oxidative stress. Elevated MDA levels (Figure 10E) further indicate membrane lipid peroxidation resulting from this oxidative burst.

Notably, the enhanced activities of antioxidant enzymes (SOD, POD, CAT, and APX) in *sig5* mutants suggest a compensatory mechanism to counter the cold-induced oxidative burst (Figure 10 F–I). This upregulation is consistent with studies showing that cold stress upregulates antioxidant systems as part of the acclimation response [41]. However, in *sig5* mutants, this upregulation appears insufficient to prevent oxidative damage, suggesting that *SIG5*-mediated maintenance of photosynthetic electron flow is fundamental to ROS homeostasis. Previous research has established that efficient electron transport through PSII and PSI is crucial for minimizing ROS generation under stress conditions [42], and our results indicate that SIG5 is integral to this process in *Arabidopsis*.

The chlorophyll deficiency observed in *sig5* mutants under cold stress (Figure 6A) may reflect both oxidative degradation and impaired synthesis of chlorophyll-binding proteins. ROS can directly damage chlorophyll molecules and inhibit chlorophyll biosynthesis enzymes [43], while proper assembly and stability of photosynthetic complexes depend on coordinated nuclear and plastid gene expression, which requires functional sigma factors [6]. The increased non-photochemical quenching (NPQ) capacity observed in *sig5* mutants (Figure 12) likely represents an attempt to dissipate excess excitation energy. However, this mechanism alone cannot compensate for fundamental defects in photosynthetic complex maintenance in the absence of functional SIG5, consistent with reports that sustained NPQ under prolonged stress may be insufficient when not accompanied by adequate repair of the photosynthetic apparatus [44].

It is important to acknowledge that the functional evidence presented here does not demonstrate the role of *BnSIG5A* in *B. napus* cold tolerance. The *Arabidopsis* mutant analysis confirms previously reported functions of *AtSIG5* [2,20], and together with the strong cold-induction of *BnSIG5A* and its chloroplast localization supports the hypothesis that *BnSIG5A* may play a similar role. However, direct validation in *B. napus* is essential for two key reasons.

First, the allotetraploid genome of *B. napus* contains four *SIG5* homologs (*BnSIG5A-D*), whereas *Arabidopsis* has only one. Our expression profiling revealed that these paralogs exhibit distinct temporal dynamics under cold stress (Figure 6 and Figure 8), suggesting possible divergence in regulatory control or functional specialization. Whether these paralogs have redundant, overlapping, or distinct functions remains unknown and requires investigation through *B. napus*-specific approaches.

Second, as a major oilseed crop cultivated across diverse climatic zones, understanding the molecular basis of cold tolerance in *B. napus* has direct agricultural applications. Natural variation in *BnSIG5* expression among cultivars may contribute to adaptation gradients.

Therefore, the current study should be viewed as a comprehensive genomic resource that identifies *BnSIG5A* as a high-priority candidate for future functional studies. Subsequent investigations should employ *B. napus*-specific approaches, including: (1) CRISPR/Cas9-mediated mutagenesis of individual *BnSIG5* paralogs; (2) stable overexpression of *BnSIG5A* in *B. napus*; (3) complementation of *Arabidopsis sig5* mutants with *BnSIG5A*; and (4) analysis of natural variation in *BnSIG5A* expression among cultivars with contrasting cold tolerance. The genomic resources provided here will facilitate such investigations.

## 4. Materials and Methods

### 4.1. Plant Materials and Treatments

Uniform and healthy seeds of the winter rapeseed cultivar ‘ZD622’ were used for this study. The seeds were cultured in a quarter-strength Hoagland’s nutrient solution in a box. After germination, the uniform-sized seedlings were shifted to half-strength Hoagland nutrient solution in plastic pots. After two weeks, the seedlings were used for cold treatment (4 °C). Following the treatments, leaf samples were taken at 0, 6, 12, 24 and 48 h and three biological replications of each treatment were included in the samples, which were thereafter instantly frozen in liquid nitrogen and then stored at −80 °C.

The *Arabidopsis thaliana* T-DNA insertion mutant lines sig5-3 (SALK_141383C) and sig5-7 (SALK_101921C) in the Col-0 background were originally obtained from the Arabidopsis Biological Resource Center (ABRC, Columbus, OH, USA; https://abrc.osu.edu/; accessed on 12 May 2025). Seeds were propagated and verified in our laboratory and subsequently made available through the material-sharing platform https://www.arashare.cn (accessed on 12 May 2025). For this study, seeds were obtained from this platform, and genotype confirmation was performed by PCR-based genotyping as described previously [2] (Supplementary Figure S1). Seeds were surface-sterilized and sown on half-strength Murashige and Skoog (MS) basal salts mixture (Duchefa Biochemie, Haarlem, The Netherlands) in 0.8% (*w*/*v*) agar at pH 5.8. Following stratification in darkness at 4 °C for 2 days, seedlings were transferred to Panasonic GXZ-450D plant growth chambers (Ningbo Jiangnan Instrument Factory, Ningbo, China). Wild type Col-0 was used as a control. Plants were cultivated under 12 h light/12 h dark cycles at 22 °C with a light intensity of 90 µmol m^−2^ s^−1^ (white light). Experimental treatments were initiated at one week and 20 days after germination. Then, seedlings were moved to an incubator at 4 °C, with all other conditions remaining unchanged.

### 4.2. Identification and Bioinformatics Analysis of BnSIG Genes

The detailed methodology for identification and bioinformatics analysis of *BnSIG* genes is provided in Supplementary File S1.

### 4.3. Phylogenetic Analysis and Chromosomal Locations

The SIG protein sequences from *A. thaliana*, *B. rapa*, *B. oleracea*, and *B. napus* were obtained from the TAIR (https://www.arabidopsis.org/) and BnIR (https://yanglab.hzau.edu.cn/) data bases. Multiple sequence alignments were carried out using MUSCLE implemented in MEGA, and the resulting files were saved in MEGA format. Phylogenetic analysis was performed in MEGA6 using the maximum likelihood (ML) method with 1000 bootstrap replicates. The phylogenetic tree was subsequently visualized and refined with Chiplot (https://www.chiplot.online/normalTree.html, accessed on 12 May 2025). Chromosomal positions of *BnSIG* genes were extracted from the GFF3 annotation file of the *B. napus* ZS11 genome and mapped using TBtools v2.450.

### 4.4. Gene Structure, Motif and Cis-Element Analysis

Cis-acting regulatory elements in promoter regions were predicted using the PlantCARE database (http://bioinformatics.psb.ugent.be/webtools/plantcare/html/, accessed on 12 May 2025). It is important to note that these predictions are based on sequence homology to known motif patterns and represent putative regulatory elements. Experimental validation (e.g., promoter-reporter assays, EMSA, ChIP) is required to confirm actual transcription factor binding and functional regulatory activity. The detailed methodology for gene structure, motif and cis-element analysis is provided in Supplementary File S1.

### 4.5. RNA Extraction and qRT-PCR Analysis

*Arabidopsis* wild type and mutant plants at 20 days after germination were subjected to low-temperature treatment for 14 days. After treatment, leaf samples were collected for subsequent analysis. After sample collection, they were immediately transferred into liquid nitrogen to ensure efficient freezing and grinding. Subsequently, reagent kits were utilized to conduct RNA extraction and reverse transcription (Supplementary File S1). For RT-qPCR testing, the obtained cDNA was diluted and utilized as a template (Supplementary File S1). Utilizing the 2^−ΔΔCt^ method, gene expression levels were determined. The primers used in the investigation are listed in Supplementary Table S4.

### 4.6. RNA-Seq Data Acquisition and Expression Analysis

#### 4.6.1. Tissue-Specific Expression Analysis

Genome-wide expression profiles for *BnSIG* genes across different tissues and developmental stages were retrieved from the *Brassica napus* Transcriptome Information Resource (BnTIR; http://yanglab.hzau.edu.cn/BnTIR, accessed on 12 May 2025) [45]. This database compiles RNA-seq data from multiple studies including root, stem, leaf, flower, silique, and seed tissues at various developmental stages. For our analysis, we extracted fragments per kilobase of transcript per million mapped reads (FPKM) values for all identified *BnSIG* genes from the following tissue samples: root, stem, leaf, flower bud, open flower, silique, and seed (at 10, 20, 30, and 40 days after pollination). Samples with three biological replicates were selected where available.

#### 4.6.2. Cold Stress Expression Analysis

To examine *BnSIG* gene expression under low-temperature stress, we analyzed publicly available RNA-seq data from the NCBI Sequence Read Archive (SRA) under accession numbers SRR7764353, SRR7764354, and SRR7764358. These datasets correspond to a published study [2] examining *B. napus* (cultivar ‘ZS11’) response to cold stress. Experimental conditions were as follows: three-week-old seedlings grown under controlled conditions (22 °C, 16 h light/8 h photoperiod, 120 µmol m^−2^ s^−1^ light intensity) were transferred to 4 °C for cold treatment. Leaf samples were collected at 0 h (control), 1 h, 2 h, 6 h, 12 h, and 24 h after treatment initiation, with three biological replicates per time point.

#### 4.6.3. Data Processing and Visualization

Raw RNA-seq reads were quality-filtered and aligned to the *B. napus* ‘ZS11’ reference genome using HISAT2. Gene expression levels were quantified as FPKM values using StringTie. For visualization, FPKM values were log2-transformed (log2 [FPKM + 1]) to normalize distribution. Heatmaps were generated using the R package pheatmap (version 1.0.12) with hierarchical clustering based on Euclidean distance. Genes with FPKM < 1 across all samples were considered not expressed and were filtered from visualization. All processed expression data used in this study are provided in Supplementary Table S5.

#### 4.6.4. Statistical Criteria

For identification of differentially expressed genes under cold stress, we applied the following criteria: |log2 fold change| ≥ 1 and false discovery rate (FDR) ≤ 0.05, as calculated by DESeq2. Time points were compared against the 0 h control. Only genes meeting these criteria were considered significantly responsive to cold treatment.

### 4.7. Subcellular Localization Analysis of SIG5-GFP Protein

The *BnSIG5A* CDS without a stop codon was amplified and ligated with homologous arms for insertion into a pCAMBIA1300 transient expression vector. A GFP fluorescent label was present after the insertion site. Agrobacterium GV3101 was transformed with either the recombinant vector or the empty vector (pCAMBIA1300-sGFP) and injected into the leaves of 5-week-old tobacco plants. Laser scanning confocal microscopy (Zeiss LSM 880, Carl Zeiss AG, Oberkochen, Germany) was used to observe GFP signals after 48 h of incubation under low light.

### 4.8. Low-Temperature Tolerance Assay of Yeast Transformants

The BY4741 yeast strain, transformed with either the pYES2-*BnSIG5A* recombinant plasmid or the empty pYES2 vector (confirmed positive), was induced with galactose. Subsequently, the cultures were serially diluted (undiluted, 10^−1^, 10^−2^, 10^−3^, and 10^−4^), and 5 μL aliquots from each dilution were spotted onto SG-U solid medium containing 2% for 48 h. The growth differences between the two strains were assessed. Yeast cultures incubated at 29 °C for 48 h served as the control for normal growth conditions.

### 4.9. Chlorophyll Measurement and Gas Exchange Parameters

The detailed methodology for the chlorophyll analysis is provided in Supplementary File S1 [46,47,48].

### 4.10. Histochemical Analysis

The detailed methodology for the histochemical analysis is provided in Supplementary File S1 [49].

### 4.11. Analysis of ROS and Antioxidant Enzymes

The detailed methodology for the analysis of ROS and antioxidant enzymes is provided in Supplementary File S1 [50,51,52].

### 4.12. Statistical Analysis

Data analysis for this study was executed using GraphPad Prism 9, TBtools v2.019, and Excel 2016. Three biological replicates were used for each experiment, and the values listed in Section 2 represent the means of those replicates plus standard error (SE). To identify significant differences between the means of treatments, one- and two-way analysis of variance (ANOVA) was performed.

## 5. Conclusions

This study provides the first comprehensive genome-wide analysis of the sigma factor gene family in *B. napus*, identifying 23 *BnSIG* genes phylogenetically classified into six distinct groups. Our analyses reveal that the family expanded primarily through segmental duplication events and was under strong purifying selection, highlighting the evolutionary constraint on these essential regulatory proteins. Promoter cis-element analysis identified cold-responsive elements (LTR and MYB motifs) enriched in several *BnSIG* genes, particularly *BnSIG5A*. Expression profiling using RNA-seq and qRT-PCR validation demonstrated that *BnSIG5* subfamily members, especially *BnSIG5A*, are strongly induced by cold stress, and subcellular localization confirmed chloroplast targeting of BnSIG5A. These results identify *BnSIG5A* as a strongly cold-responsive candidate gene in *B. napus*. Analysis of *Arabidopsis* SIG5 T-DNA insertion mutants confirmed previously reported functions of *AtSIG5* in cold tolerance [2], including roles in maintaining photosynthetic integrity and mitigating oxidative stress. Based on the sequence homology, cold-inducible expression, and chloroplast localization of *BnSIG5A*, we hypothesize that *BnSIG5A* may play a conserved role in cold stress responses. However, direct validation in *B. napus* through approaches such as CRISPR/Cas9-mediated mutagenesis or stable overexpression is required to test this hypothesis definitively. This work establishes a valuable genomic framework for the *SIG* family in *B. napus* and provides a foundation for future functional studies aimed at understanding cold tolerance in this economically important crop species.

## Data Availability

All data presented in this study are available in the article and Supplementary Materials.

## Supplementary Materials

The following supporting information can be downloaded at: https://www.mdpi.com/article/10.3390/ijms27073010/s1.
