# Supplementary material for: Genome-Wide Identification of Sigma Factors in Brassica napus and Role of BnSIG5A in Response to Cold Stress
Source: Int J Mol Sci. 2026 Mar 26;27(7):3010. doi: 10.3390/ijms27073010 (PMC13073550; doi:10.3390/ijms27073010)
Supplement: Supplementary file 1 [file ijms-27-03010-s001.zip › Supplementary file S1.pdf]

Supplementary file S1

1. Identification and bioinformatics analysis of SIG genes

Six *A. thaliana* SIG protein sequences were retrieved from the Arabidopsis database (https://www.arabidopsis.org/), together with genomic data of *B. napus* from the *B. napus* database (https://yanglab.hzau.edu.cn/). The hidden Markov model (HMM) profile of the SIG-type DBD domain (PF0454) was obtained from the Pfam database (https://www.ebi.ac.uk/interpro/entry/pfam) and used for initial screening with HMMER. Candidate SIG gene family members were subsequently validated for the presence of complete domains using the NCBI Conserved Domain Database (https://www.ncbi.nlm.nih.gov/cdd). Protein physicochemical properties, including sequence length, molecular weight, theoretical isoelectric point, instability index, aliphatic index, and grand average of hydropathicity, were predicted using ProtParam (https://web.expasy.org/protparam/). Transmembrane features, subcellular localization, and secondary structure were further assessed with TMHMM 2.0 (https://services.healthtech.dtu.dk/services/TMHMM-2.0/), Cell-PLoc (http://www.csbio.sjtu.edu.cn/bioinf/Cell-PLoc-2/), and SOPMA (https://npsa.lyon.inserm.fr/cgi-bin/npsa_automat.pl?page=/NPSA/npsa_sopma.html), respectively.

2. Gene structure, motif and cis-element analysis

The exons and introns of each *SIG* gene were obtained from the white clover genome annotation file. Conserved protein motifs were identified with the MEME Suite (https://meme-suite.org/) using parameters set to a maximum of 10 motifs and an optimal width of 5–200 amino acids. Gene structures, motif distributions, and protein domains were visualized and analyzed in TBtools. Cis-regulatory elements located within 2,000 bp upstream of the transcription start site of each SIG gene were predicted using the PlantCARE database (http://bioinformatics.psb.ugent.be/webtools/plantcare/html/).

3. Chlorophyll measurement and gas exchange parameters

Chlorophyll pigments were extracted from 0.2 g of fresh, fully expanded leaves using 80% acetone at room temperature. The extracts were centrifuged at 2800 × g for 15 min, and the resulting supernatants were collected for absorbance measurements at 750, 663, 652, 645, and 470 nm using a Hitachi F-4600 fluorescence spectrophotometer [1,2].

Photosystem II (PSII) efficiency was assessed by chlorophyll fluorescence imaging using an IMAGING-PAM M-series MAXI system (Walz). Twenty-day-old seedlings grown at 25 °C were transferred to 4 °C for two weeks prior to measurement. For all assays, plants were dark-adapted for 20 min before fluorescence recording. Measurements were initiated with low-frequency measuring light pulses (1 Hz, intensity 3), followed by a saturating pulse. Chlorophyll fluorescence of mature rosette leaves was additionally measured with a MINI-PAM fluorometer (Walz) after 20 min dark adaptation [3].Leaf gas exchange analysis

The net photosynthetic rate, stomatal conductance, and transpiration rate of fully expanded leaves were measured between 09:00 and 11:00 h using a portable photosynthesis system (LI-6400XT, LI-COR Inc., Lincoln, NE, USA). Measurements were taken at a photon flux density of 1000 μmol m^{-2} s^{-1}, a chamber flow rate of 500 μmol s^{-1}, a leaf temperature of 28 °C, and an ambient CO_2 concentration of 380 μmol mol^{-1}, while maintaining a vapor pressure deficit of 2.0 kPa [4]. Each treatment was represented by four biological replicates.

4. Histochemical analyses

The production of superoxide (O^{2-}) and hydrogen peroxide (H_2O_2) in plant tissues was visualized by staining with nitroblue tetrazolium (NBT) and 3, 3′-diaminobenzidine (DAB), respectively. Leaves were excised and submerged in 10 mM PBS (pH 7.8) containing 0.1 % (w/v) NBT or 1 mg mL^{-1} DAB (pH 3.8). The leaves were then left to incubate for a full day at room temperature in the dark. After staining, the leaves were rinsed with deionized water and decolorized with 95 % (v/v) ethanol at 95 °C for 10 min. The development of brown (DAB) or blue (NBT) precipitates in the leaf tissues was a sign that ROS had accumulated[5].

5. Analysis of ROS and antioxidant enzymes

Membrane lipid peroxidation was estimated by quantifying malondialdehyde (MDA) concentration [5]. Hydrogen peroxide (H_2O_2) content was measured using the method described in previous studies [6]. For the estimation of antioxidant enzyme activities, 0.5 g of leaf tissue was homogenized in 10 mL of 50-mM phosphate buffer and centrifuged at 13,000 rpm for 10 minutes. The capacity of superoxide dismutase (SOD) to prevent NBT photochemical degradation was measured by using spectrophotometer [7]. Guaiacol peroxidase (POD) activity was assessed as described in previous studies [8]. Catalase (CAT) activity was determined in previous studies 41. Ascorbate peroxidase (APX) activity was measured by the rate of H_2O_2-dependent oxidation of ascorbic acid, as described by previous studies [7].

6. RNA extraction and qRT-PCR analysis

Total RNA was isolated from leaf tissues of B.napus under both normal and cold conditions using the TRIzol reagent (Invitrogen, USA). Each tissue sample was collected from five plants, with three biological replicates prepared for each tissue type. Following RNA quality assessment, libraries were constructed. Messenger RNAs (mRNAs) were used as templates for reverse transcription into complementary DNAs (cDNAs), which were then puriffed and used to construct cDNA libraries. Quantitative PCR (RT-qPCR) was performed and the analysis was conducted using SYBR Green Master Mix (Takara, Japan) and a CFX96 Touch Real-Time PCR Detection System (Bio-Rad, USA).

7. Identification of T-DNA insertion mutants

The Arabidopsis mutant lines SIG5-3 (SALK_141383C) and SIG5-7 (SALK_101921C) are SALK T-DNA insertion lines originally distributed by the Arabidopsis Biological Resource Center (ABRC, Columbus, OH, USA). The lines were obtained through arashare.cn, a legal material-sharing platform that facilitates access to ABRC resources for researchers in China. The stock numbers and original source of the mutants are consistent with the records of ABRC (https://abrc.osu.edu/). Homozygous insertion lines were isolated by PCR-based genotyping. Genomic DNA was extracted from individual T3 (or T4) plants.

The exact site of T-DNA insertion within the SIG5 locus was confirmed by PCR using a combination of gene-specific primers and a T-DNA border primer (Suplementary Table S4). During PCR amplification, the LP and RP primer pair amplifies the wild-type genomic region, while the BP and RP primer pair amplifies the T-DNA insertion. Because the T-DNA fragment is several kilobases long, the LP+RP primers cannot successfully amplify the target in T-DNA insertion mutants. In the wild type, where no T-DNA is inserted, the BP+RP primers also fail to amplify any product. Therefore, in the wild type, only the LP+RP PCR product is present; in the mutant, only the BP+RP PCR product is present; and in the heterozygote, both bands appear. We can get the detail information of the precise insertion positions of mutants from http://signal.salk.edu/cgi-bin/tdnaexpress. (Fig. S1).

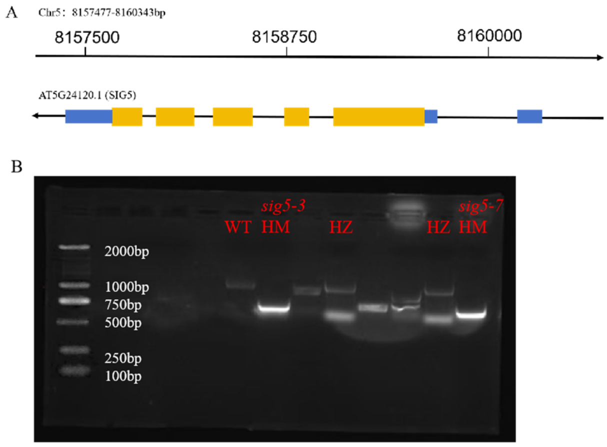

Figure S1 A. Gene structure of *AtSIG5* Locus: Chr5 8157477-8160343 ; B. Validation of the *Arabidopsis* T-DNA insertion mutants, WT(wide type), HM (homozygous), HZ (heterozygous).

8. qRT-PCR of genes expression related to RuBP hydroxylase large subunit synthesis and the psbI, psbK, psbD, psbC

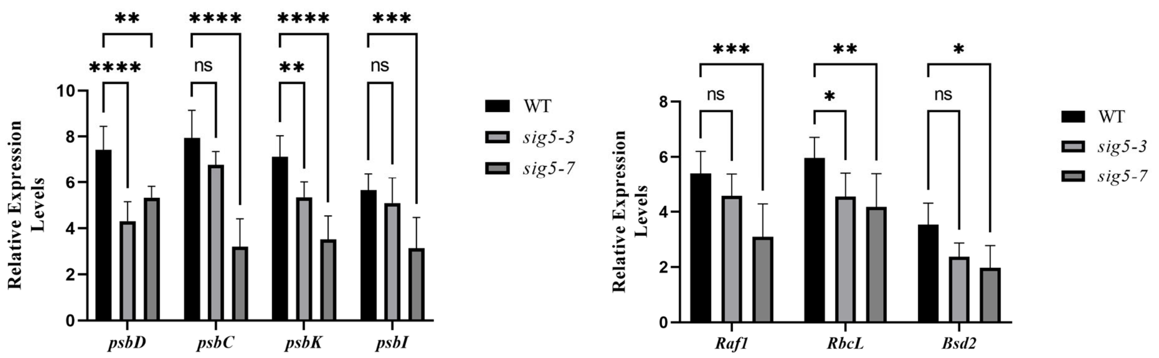

Figure S2 Expression analysis of genes related to RuBP hydroxylase large subunit synthesis and the psbI, psbK, psbD, psbC in the nomal condition by RT-qPCR. The 2-ΔΔCt method was employed to assess gene expression patterns relative to Actin, based on three replicates. The results are presented as the mean ± standard deviation. Statistical significance was evaluated using Student's t-test, WT serves as control, with significance levels indicated as follows: * = 0.05, ** = 0.01, *** = 0.001, **** = 0.0001.

Reference

1. Porra, R. J.; Thompson, W. A.; Kriedemann, P. E. Determination of accurate extinction coefficients and simultaneous equations for assaying chlorophylls a and b extracted with four different solvents: verification of the concentration of chlorophyll standards by atomic absorption spectroscopy. BBA-Bioenerg. 1989, 975, 384－394.

2. Lichtenthaler, H. K.; Wellburn, A. R. Determinations of total carotenoids and chlorophylls a and b of leaf extracts in different solvents. Biochem. Soc. Trans. 1983, 11, 591－592.

3. Cano-Ramirez, D. L.; Panter, P. E.; Takemura, T.; de Fraine, T. S.; de Barros Dantas, L. L.; Dekeya, R.; Barros-Galvão, T.; Paajanen, P.; Bellandi, A.; Batstone, T.; Manley, B. F.; Tanaka, K.; Imamura, S.; Franklin, K. A.; Knight, H.; Dodd, A. N. Low-temperature and circadian signals are integrated by the sigma factor SIG5. Nat. Plants 2023, 9(4), 661－672.

4. Parsons, R.; Wevers, J.; Lawson, T.; Godber, L. Rapid and straightforward estimates of photosynthetic characteristics using a portable gas exchange system. Photosynthetica 1998, 34(2), 265 – 279.

5. Kuk, Y. I.; Shin, J. S.; Burgos, N. R.; Hwang, T. E.; Han, O.; Cho, B. H.; Jung, S.; Guh, J. O. Antioxidative enzymes offer protection from chilling damage in rice plants. Crop Sci. 2003, 43, 2109 – 2117.

6. Sergiev, I.; Alexieva, V.; Karanov, E. Effect of spermine, atrazine, and combination between them on some endogenous protective systems and stress markers in plants. Compt. Rend. Acad. Bulg. Sci. 1997, 51, 121 – 124.

7. Nakano, Y.; Asada, K. Hydrogen peroxide is scavenged by ascorbate-specific peroxidase in spinach chloroplasts. Plant Cell Physiol. 1981, 22, 867 – 880.

8. Aebi, H. Catalase in vitro. In: Methods in Enzymology; Elsevier: 1984; pp 121 – 126.
