# Supplementary material for: Genome-Wide Identification of Sigma Factors in Brassica napus and Role of BnSIG5A in Response to Cold Stress
Source: Int J Mol Sci. 2026 Mar 26;27(7):3010. doi: 10.3390/ijms27073010 (PMC13073550; doi:10.3390/ijms27073010)
Supplement: Supplementary file 1 [file ijms-27-03010-s001.zip › Supplementary Table S1-S4.pdf]

Supplementary Table S1-S4

Supplementary Table S1 Molecular characteristics of *BnSIG* genes in *B.napus.*

	Sequence ID	Number of Amino Acid	Molecular Weight	Theoretical pI	Instability Index	Aliphatic Index	Grand Average of Hydropathicity
BnSIG4A	BnaA02T0050300ZS	408	46212.45	9.71	65.76	90.51	-0.458
BnSIG1A	BnaA02T0247900ZS	492	55730.68	9.45	43.89	89.37	-0.5
BnSIG6A	BnaA03T0409200ZS	88	10332.18	10.38	41.6	99.66	-0.241
BnSIG3A	BnaA04T0052200ZS	684	77541.56	9.59	43.46	81.99	-0.415
BnSIG6B	BnaA04T0237300ZS	558	62958	9.63	57.13	85.11	-0.553
BnSIG2A	BnaA06T0051100ZS	595	66818.85	9.32	49.33	72.5	-0.659
BnSIG2B	BnaA06T0226400ZS	258	28280.89	6.47	55.23	76.12	-0.426
BnSIG5A	BnaA06T0326400ZS	510	58235.64	9.99	46.14	89.06	-0.587
BnSIG2C	BnaA08T0299000ZS	572	64117.03	9.44	49.26	76.91	-0.585
BnSIG5B	BnaA09T0068800ZS	501	57350.76	10.02	45.4	92.59	-0.545
BnSIG4B	BnaA10T0219700ZS	417	46739.59	9.76	63.45	88.13	-0.476
BnSIG4C	BnaC02T0058000ZS	409	45995	9.83	67.88	88.63	-0.473
BnSIG1B	BnaC02T0332000ZS	481	54479.41	9.54	42.09	90.42	-0.484
BnSIG6C	BnaC03T0206900ZS	149	17169.99	9.8	44.07	94.16	-0.251
BnSIG6D	BnaC03T0207000ZS	368	42052.61	8.99	59.17	85.08	-0.205
BnSIG3B	BnaC04T0330300ZS	564	64140.12	9.84	48.85	80.21	-0.465
BnSIG6E	BnaC04T0552800ZS	568	64043.43	9.54	54.39	87.38	-0.507
BnSIG2D	BnaC05T0063700ZS	583	65381.11	9.24	50.06	72.81	-0.665
BnSIG5C	BnaC07T0367800ZS	561	63786.01	9.9	45.72	89.64	-0.549
BnSIG2E	BnaC08T0184400ZS	571	63946.76	9.44	46.56	77.04	-0.584
BnSIG5D	BnaC09T0058900ZS	503	57454.76	9.88	48.78	90.85	-0.543
BnSIG4D	BnaC09T0516800ZS	232	26738.66	10.02	78.89	79.83	-0.708
BnSIG4E	BnaC09T0523900ZS	419	46985.92	9.76	65.08	88.19	-0.461

Supplementary Table S2 Molecular characteristics of BnSIG proteins in *B.napus*.

Protein	Alpha helix (Hh)(%)	Extended strand (We)(%)	Beta bridge (aa)(%)	Random coil (aa)(%)	Subcellular Location
BnSIG4A	267 (65.44%)	40 (9.80%)	0 (0.00%)	101 (24.75%)	Chloroplast. Cytoplasm. Mitochondrion.
BnSIG1A	318 (64.63%)	46 (9.35%)	0 (0.00%)	128 (26.02%)	Nucleus
BnSIG6A	58 (65.91%)	9 (10.23%)	0 (0.00%)	21 (23.86%)	Chloroplast. Golgi apparatus. Nucleus.
BnSIG3A	419 (61.26%)	68 (9.94%)	0 (0.00%)	197 (28.80%)	Chloroplast.
BnSIG6B	368 (65.95%)	26 (4.66%)	0 (0.00%)	164 (29.39%)	Cytoplasm. Nucleus.
BnSIG2A	382 (64.20%)	48 (8.07%)	0 (0.00%)	165 (27.73%)	Chloroplast. Cytoplasm. Nucleus.
BnSIG2B	146 (56.59%)	22 (8.53%)	0 (0.00%)	90 (34.88%)	Nucleus
BnSIG5A	349 (68.43%)	42 (8.24%)	0 (0.00%)	119 (23.33%)	Chloroplast. Cytoplasm. Mitochondrion. Nucleus.
BnSIG2C	400 (69.93%)	37 (6.47%)	0 (0.00%)	135 (23.60%)	Chloroplast. Mitochondrion. Nucleus.
BnSIG5B	342 (68.26%)	40 (7.98%)	0 (0.00%)	119 (23.75%)	Chloroplast.
BnSIG4B	282 (67.63%)	34 (8.15%)	0 (0.00%)	101 (24.22%)	Chloroplast. Cytoplasm.
BnSIG4C	271 (66.26%)	29 (7.09%)	0 (0.00%)	109 (26.65%)	Cytoplasm. Mitochondrion.
BnSIG1B	313 (65.07%)	43 (8.94%)	0 (0.00%)	125 (25.99%)	Cytoplasm.
BnSIG6C	83 (55.70%)	13 (8.72%)	0 (0.00%)	53 (35.57%)	Cytoplasm. Golgi apparatus. Nucleus.
BnSIG6D	260 (70.65%)	30 (8.15%)	0 (0.00%)	78 (21.20%)	Chloroplast. Nucleus.
BnSIG3B	383 (67.91%)	44 (7.80%)	0 (0.00%)	137 (24.29%)	Cytoplasm.
BnSIG6E	399 (70.25%)	22 (3.87%)	0 (0.00%)	147 (25.88%)	Cytoplasm. Nucleus.
BnSIG2D	410 (70.33%)	40 (6.86%)	0 (0.00%)	133 (22.81%)	Chloroplast. Mitochondrion. Nucleus.
BnSIG5C	359 (63.99%)	47 (8.38%)	0 (0.00%)	155 (27.63%)	Chloroplast. Nucleus.
BnSIG2E	386 (67.60%)	43 (7.53%)	0 (0.00%)	142 (24.87%)	Chloroplast. Nucleus.
BnSIG5D	359 (71.37%)	40 (7.95%)	0 (0.00%)	104 (20.68%)	Chloroplast. Cytoplasm. Mitochondrion. Nucleus.
BnSIG4D	166 (71.55%)	14 (6.03%)	0 (0.00%)	52 (22.41%)	Chloroplast.
BnSIG4E	273 (65.16%)	26 (6.21%)	0 (0.00%)	120 (28.64%)	Cytoplasm. Nucleus.

Supplementary Table S3 Ka_Ks analysis of gene pairs in *BnSIG* gene duplicated events

Seq_1	Seq_2	Ka	Ks	Ka_Ks	Effective Length	Purpfying selection	Duplication type
BnSIG4A	BnSIG4B	0.061564322	0.347695968	0.17706367	1215	Yes	Segmental
BnSIG4A	BnSIG4C	0.022276176	0.113317675	0.19658165	1224	Yes	Segmental
BnSIG1A	BnSIG1B	0.005425796	0.024404797	0.22232499	1443	Yes	Segmental
BnSIG4A	BnSIG4E	0.060622044	0.333054932	0.18201816	1221	Yes	Segmental
BnSIG6B	BnSIG6C	0.109189929	0.491183568	0.22229964	426	Yes	Segmental
BnSIG6B	BnSIG6E	0.012623572	0.087650926	0.14402098	1665	Yes	Segmental
BnSIG3A	BnSIG3B	0.022091429	0.123863268	0.17835335	1653	Yes	Segmental
BnSIG2A	BnSIG2C	0.058120171	0.343306364	0.16929535	1695	Yes	Segmental
BnSIG5A	BnSIG5B	0.052968529	0.335353353	0.15794841	1482	Yes	Segmental
BnSIG2A	BnSIG2D	0.024661867	0.154960687	0.15914919	1719	Yes	Segmental
BnSIG5A	BnSIG5C	0.011166193	0.154989381	0.07204489	1530	Yes	Segmental
BnSIG2A	BnSIG2E	0.055661004	0.330709699	0.16830775	1695	Yes	Segmental
BnSIG5A	BnSIG5D	0.048670615	0.367778048	0.13233692	1482	Yes	Segmental
BnSIG2C	BnSIG2D	0.055273623	0.371022181	0.1489766	1710	Yes	Segmental
BnSIG2C	BnSIG2E	0.010980555	0.140642883	0.07807402	1710	Yes	Segmental
BnSIG5B	BnSIG5C	0.049908802	0.364518264	0.13691715	1482	Yes	Segmental
BnSIG5B	BnSIG5D	0.013986419	0.122400387	0.11426777	1500	Yes	Segmental
BnSIG4B	BnSIG4C	0.068661468	0.369726005	0.18570906	1215	Yes	Segmental
BnSIG4B	BnSIG4E	0.012839115	0.143981241	0.08917214	1248	Yes	Segmental
BnSIG4C	BnSIG4E	0.067080642	0.341058565	0.19668365	1221	Yes	Segmental
BnSIG6C	BnSIG6E	0.10758785	0.561618453	0.19156751	426	Yes	Segmental
BnSIG2D	BnSIG2E	0.053783415	0.357742669	0.15034107	1710	Yes	Segmental
BnSIG5C	BnSIG5D	0.045656936	0.427936766	0.10669085	1482	Yes	Segmental

Supplementary Table S4 All primers were used in this study.

Primers used for quantitative real time PCR

Primer name	Primer sequence (5’ to 3’)
BnSIG1A	F:CTCGGCAAAGGAGAATGGGT
	R:TTTTCTTGGACAAGCGCACG
BnSIG1B	F:TGTCTCTGCCTCTCCTCTCT
	R:CAGTCTCGATGCCTCAGGAT
BnSIG5A	F:GAAGGAGCCACGTGTTGATC
	R:AGCACTGATCTTCGGACACA
BnSIG5B	F:TGGGAAAGGAGACAGGACAC
	R:CGGTTCAGTTCGGTTTGGTT
BnSIG5C	F:TAACTTCACTCGCGTCCCTT
	R:TGCTCTCAAGACTTCACGGT
BnSIG5D	F:GGTCTTCGGCTCGTCAAATC
	R:CAAGCTTCCACGTTCGAACA
psbD	F:ACGTCTTGGAGCTAACGTGG
	R:CGCAGATCCCAAAAACGCAT
psbC	F:ACGTCTTGGAGCTAACGTGG
	R:TTTTTCAAATCCCGCTGCCG

psbk	F:AGAGGCCTACGCCTTTTTGA<br>R:ACTTACAGCGGCTTGCCAAA
psbL	F:TGACACAATCAAATCCGAACGA<br>R:AGTAATAACCCCCAATAGAGACTGG
Raf1	F:TTCACCAACCCGATTACCCG<br>R:CCAACCATCCAACGTCTCCA
RbcL	F:GCATATGCCTGCTTTGACCG<br>R:GGGTGGCCTAAAGTTCCTCC
Bsd2	F:GCACTCTGTTGGCTTTGCAG<br>R:GGTGCTCAAGAACCCACCAA
**Primers used to amplify CDS of BnSIG5A for subcellular localization**	
BnSIG5A	F:gagaacacgggggacgagctcATGGGAGTCGTGTTTATTTCAAGTT<br>R:gctcaccatgtcgactctagGATGATGTACTGACGAAGGTAATCAAC
**Primers used for T-DNA mutant identification**	
sig5-3	LP:TCTCATACCCGCTTGACAAAG<br>RP:GTTCAGCTGCAAGATCTCCAC
sig5-7	LP:CCATTCTCTAGTGTCAGCCAC<br>RP:GTTTGAGATGGGAAGACCTCC
BP	ATTTTGCCGATTTCGGAAC
**Primers used for CDS of BnSIG5A for yeast transformants**	

BnSIG5A-Y	F:attaagcttggtaccgagctcATGGGAGTCGTGTTTATTTCAAGTT<br>R:attaagcttggtaccgagctcATGGGAGTCGTGTTTATTTCAAGTT
